# Data-driven non-intrusive reduced order modelling of selective laser melting additive manufacturing process using proper orthogonal decomposition and convolutional autoencoder

**DOI:** 10.1186/s40323-025-00305-6

**Published:** 2025-08-05

**Authors:** Shubham Chaudhry, Azzedine Abdedou, Azzeddine Soulaïmani

**Affiliations:** Department of Mechanical Engineering, École de Technologie Supérieure, Montréal, QC H3C 1K3 Canada

**Keywords:** Reduced-order model, Convolutional autoencoder, Additive manufacturing, Deep learning, Proper orthogonal decomposition

## Abstract

This study proposes and compares two data-driven, non-intrusive reduced-order models (ROMs) for additive manufacturing (AM) processes: a combined proper orthogonal decomposition-artificial neural network (POD-ANN) and a convolutional autoencoder-multilayer perceptron (CAE-MLP). The POD-ANN model utilizes proper orthogonal decomposition to create a reduced-order model, which is then combined with an artificial neural network to establish a surrogate model linking the snapshot matrix to the input parameters. This approach effectively reduces the dimensionality of the high-fidelity snapshot matrix and constructs a regression framework for accurate predictions. Conversely, the CAE-MLP model employs a 1D convolutional autoencoder to reduce the spatial dimension of a high-fidelity snapshot matrix derived from numerical simulations. The compressed latent space is then projected onto the input variables using a multilayer perceptron (MLP) regression model. This method leverages deep learning techniques to handle the complexity of the data and improve prediction accuracy. The accuracy and efficiency of both models are evaluated through thermo-mechanical analysis of an AM-built part. The comparison of statistical moments from high-fidelity simulation results with ROM predictions reveals a strong correlation. Furthermore, the predictions are validated against experimental results at various locations. While both models demonstrate good agreement with experimental data, the CAE-MLP model outperforms the POD-ANN model in terms of prediction accuracy and performance. The findings highlight the potential of integrating reduced-order modeling techniques with machine learning algorithms to enhance the analysis of complex AM processes. The proposed models offer a robust framework for future research and applications in the field of additive manufacturing, providing high precision and efficiency.

## Introduction

Laser Powder Bed Fusion (L-PBF) is a cutting-edge Additive Manufacturing (AM) technology that fabricates 3D parts by fusing successive layers of powder using a high-energy laser beam. L-PBF comprises two primary techniques: Electron Beam Melting (EBM), which uses a high-energy electron beam, and Selective Laser Melting (SLM), which employs a high-power laser to bond powder layers. This process enables the production of complex, robust geometries with minimal material waste, significantly reducing manufacturing costs. Despite these advantages, L-PBF faces challenges such as part distortion, dimensional inaccuracies, and premature build failures. This study focuses specifically on the SLM process, analyzing temperature evolution and strain in the fabricated part to improve understanding of its thermal and mechanical behavior during manufacturing.

In SLM, steep thermal gradients occur between the melt pool (reaching several thousand degrees Celsius) and surrounding solidified regions, which remain near ambient or preheat temperatures. These gradients induce residual stresses that can cause deformation and incompatibility. The combination of extreme temperature differences and a rapidly moving high-power laser requires highly nonlinear, finely meshed numerical models for accurate simulation. Moreover, SLM involves multiple length and time scales, further complicating numerical analyses. Rapid temperature changes in the melt pool occur within milliseconds to seconds, while the layer-by-layer building process may extend over hours or even days, with prolonged cooling periods. Phase transformations and latent heat during the powder-to-liquid-to-solid transitions add to the complexity and computational cost of thermal analysis. As a result, there is an urgent need for advanced numerical methods or alternative approaches to reduce these computational demands.

Data-driven machine learning models offer a promising alternative for studying the SLM process [1, 2]. Ravichander et al. [3] developed a neural network model to predict SLM outputs, training on experimental data and applying the model to generate new datasets. Similarly, Chaudhry et al. [4] introduced an ML framework to construct a surrogate model for SLM optimization. Other studies [5–9] have also explored ML approaches for additive manufacturing. However, as dataset dimensionality grows, these methods face scalability challenges. For instance, in [4], the data matrix for training the deep neural network (DNN) had dimensions of 360 × 287. Expanding this to 360 × 97,650 by increasing input vector sizes would render the DNN approach impractical due to the explosion in trainable parameters, which can reach millions or even billions. depending on network depth and size.

Recently, reduced-order modeling (ROM) has gained popularity in the computational community as an effective way to reduce computational costs without sacrificing accuracy, especially when dealing with large datasets. ROM enables rapid surrogate models for expensive simulations, making it particularly valuable for optimization tasks, real-time tracking, and online predictions in both industrial applications and fundamental science.

One of the most widely used ROM techniques is Proper Orthogonal Decomposition (POD). Originally introduced by Pearson [10] in 1901, POD has evolved into a robust and efficient tool for ROM analysis across various fields [11]. Recent developments have introduced non-intrusive methods for determining the coefficients of linear POD approximations through data-driven approaches, while preserving the integrity of the governing equations [12]. These include stochastic frameworks such as POD with Polynomial Chaos Expansion (POD-PCA) and POD combined with Artificial Neural Networks (POD-ANN), which build regression models linking input parameters to the coefficients of the POD basis [13–17]. Zhao et al. [18] applied POD to analyze the thermal behavior of electron beam melting (EBM) systems, using ABAQUS to investigate temperature distributions in a moving energy source. Similarly, studies [19–21] proposed POD-based ROM approaches for linear and nonlinear transient heat transfer problems, demonstrating strong agreement between standard FEM simulations and POD-FEM results.

The essence of POD lies in constructing a high-fidelity snapshot matrix to extract a small set of eigenmodes and the coefficients of a linear basis derived from these modes. Zhao et al. [22] used POD to develop a parameter map and POD bases via a regression tree method, which was later applied to predict outcomes for new sets of input variables. In related studies [14, 15], POD modes were combined with machine learning models, including artificial neural networks (ANNs). This integration of POD and ML has proven highly effective for handling large datasets and complex processes.

Recent advances in additive manufacturing have leveraged Physics-Informed Neural Networks (PINNs) to model thermal-mechanical behavior and melt-pool dynamics. Liao, et al. [23] developed a PINN-based thermal model for SLM with parameter inference, and Li et al. [24] provided one of the first 3D PINN formulations for temperature and flow prediction in SLM. More recently, Sharma et al. [19] introduced thermomechanicallyaugmented PINNs for stress prediction, and Zhu et al. [25] proposed a transfer learning-enhanced PINN (TLEPINN) for rapid meltpool morphology estimation. These studies underscore the growing integration of PINNs in additive manufacturing for realtime, highfidelity modeling and highlight future directions for extending the capabilities of data-driven reduced-order models.

Building on this context, the present study proposes a framework for non-intrusive reduced-order models that combine POD and CAEs in two distinct approaches to reduce the dimensionality of high-fidelity matrices obtained from finite element simulations. These frameworks are entirely data-driven and aim to predict strain fields in SLM-built parts efficiently while preserving accuracy. While POD’s linearity is a strength, it also limits its ability to represent complex dynamic systems [26]. It often fails to capture transient or highly localized features due to its reliance on global basis functions, which may not localize well in space or time. The quality of snapshots is another critical factor, as insufficient coverage of significant regimes can lead to inadequate reduced models. Modal truncation, often determined heuristically, introduces a trade-off between computational efficiency and the ability to capture essential dynamics. Furthermore, the orthogonality constraint of POD modes may not align with the actual physics in certain systems, such as turbulent flows, where relevant structures are not orthogonal. Although POD modes are often considered physically interpretable, this clarity can diminish in highly complex systems, making it harder to relate them to meaningful physical phenomena.

To address these limitations, nonlinear manifolds have been explored as alternatives for dimensionality reduction, with several approaches leveraging deep learning techniques [27, 28]. One such method is the autoencoder, which consists of two components: an encoder and a decoder. Jin et al. [29] introduced a convolutional neural network (CNN)-based autoencoder (CAE) model for detecting anomalies in AM-built parts. CAEs incorporate operations such as convolution, multilayer perceptron, upscaling, and pooling [30, 31], which help reduce the number of trainable parameters. However, due to their complexity, CAEs are prone to overfitting if not properly regularized. Compared to POD, the CAE-MLP model generally demands more training and prediction time; however, this trade-off is justified by its higher accuracy, especially for strongly nonlinear systems. Unlike traditional linear bases, CAEs excel at capturing complex, hierarchical, and spatially localized features. This expressive nonlinear power is essential for accurately modeling turbulent flows, highly transient phenomena, or systems where linear assumptions fail. Moreover, CAEs can generalize effectively across diverse operating conditions, potentially eliminating the need for retraining or recalibration when applied to new scenarios. Although CAEs require higher initial training costs, they enable high-fidelity surrogate predictions that can accelerate convergence in optimization loops and design studies—offsetting their slightly slower prediction times. In related studies [32–34], CAEs have been combined with Long Short-Term Memory (LSTM) networks to develop surrogate modeling frameworks, particularly for time-dependent predictions in AM-built parts.

This study proposes a framework of non-intrusive reduced-order models (ROMs) for the parametric analysis of the SLM building process. The data-driven framework integrates POD and CAEs in two distinct approaches to reduce the dimensionality of high-fidelity matrices obtained from finite element simulations. These solution matrices represent normal directional strains computed using Workbench Additive software [35]. This comparison aims to evaluate how the model architectures and training procedures influence the learned latent modes and the accuracy of reconstructions and predictions. In the first approach, POD bases of the strain matrices are computed, and a multilayer perceptron (MLP) is trained to map input variables to the POD coefficients. This surrogate model is then used to predict strains for new sets of input parameters. In the second approach, dimensionality reduction is performed using a CAE, where the spatial dimension is encoded into a latent space. An MLP maps this latent space to the input variables, while the decoder reconstructs the original spatial dimension from the latent representation. Together, the trained MLP and decoder reconstruct strain fields for new process parameters.

The proposed framework is applied to the stochastic analysis of a benchmark SLM-built part, AMB2018-1, produced by the National Institute of Standards and Technology (NIST) [36]. This work introduces new capabilities for reduced-order modeling of complex physical systems.

The paper is organized as follows: Sect. Mathematical modelling presents the mathematical background of POD and CAE. Section Results and Discussion evaluates the performance of the proposed framework through a comparison with benchmark results. Section Conclusion provides concluding remarks and directions for future research.

## Mathematical modelling

### Proper orthogonal decomposition (POD)

POD was originally developed in the study of turbulent flow fields to decompose random vector fields into deterministic functions that capture the fluctuating kinetic energy of the flow [37, 38]. The underlying idea was that a finite set of deterministic functions, known as POD modes, could effectively describe the flow structure. Since its inception, POD has been widely adopted across various scientific and engineering domains.

POD exhibits two key properties: optimality and orthogonality. Optimality ensures that the decomposition is the most efficient, with the leading modes capturing the maximum possible energy compared to any other linear decomposition of the data. Orthogonality ensures that the time series of the modal coefficients are linearly uncorrelated, a critical feature for constructing stable and accurate reduced-order models.

Let us suppose $\:Y=\left[{y}_{1},{y}_{2}\:,\:.\:.\:.\:.\:\:{y}_{n}\right]$ is a real-valued $\:m\times\:n$ matrix whose rank is $\:d\:\le\:min\left\{m,n\right\}$ with columns $\:{y}_{j}\in\:\:{R}^{m},\:\:1\le\:j\le\:n$. POD is combined with singular value decomposition (SVD) to obtain the reduced order model and a low-rank approximation that is easy to compute [38]. The SVD assures that there are real numbers $\:{\sigma\:}_{1}\:\ge\:{\sigma\:}_{2\:}\ge\:\dots\:\dots\:\dots\:{\ge\:\sigma\:}_{d}\:>0\:\:\text{a}\text{n}\text{d}\:$orthogonal matrices $\:{\Psi\:}\:\in\:\:{R}^{m\times\:m},\:\:$with columns $\:{\left\{{\psi\:}_{i}\right\}}_{i=1}^{m}$, and $\:{\Phi\:}\:\in\:\:{R}^{n\times\:n},\:\:$with columns $\:{\left\{{\varphi\:}_{\varvec{j}}\right\}}_{\varvec{j}=1}^{\varvec{n}}$, such that

1 $$\:{{\Psi\:}}^{T}Y{\Phi\:}\:=\left(\begin{array}{cc}D&\:0\\\:0&\:0\end{array}\right)$$

Here, D = diag$\:{(\sigma\:}_{1}\:,{\sigma\:}_{2\:}\dots\:\:{\sigma\:}_{d})\in\:\:{R}^{d\times\:d}$. The zero blocks in Eq. (1) have the appropriate dimensions, and T represents the matrix transpose. In addition, $\:{\left\{{\psi\:}_{i}\right\}}_{i=1}^{d}$ and $\:{\left\{{\varphi\:}_{j}\right\}}_{j=1}^{d}$ satisfy the properties

2 $$\:Y{\varphi\:}_{i}={\sigma\:}_{i}\:{\psi\:}_{i}\:\:\text{a}\text{n}\text{d}\:\:{Y}^{T}{\psi\:}_{i}={\sigma\:}_{i}{\varphi\:}_{i}\:\:\:\text{w}\text{h}\text{e}\text{r}\text{e}\:i=1,\:2\dots\:.\:d$$

which are eigenvectors of $\:Y{Y}^{T}\:$ and $\:{Y}^{T}Y$, respectively, with the eigenvalues $\:{\lambda\:}_{i}={\sigma\:}_{i}^{2}>0,\:\:i=1,\:2\dots\:.\:d$. Also, $\:{\left\{{\psi\:}_{i}\right\}}_{i=d+1}^{m}$and $\:{\left\{{\varphi\:}_{j}\right\}}_{j=d+1}^{n}$are eigenvectors with the eigenvalue 0 of $\:Y{Y}^{T}\:$ and $\:{Y}^{T}Y$ (if $\:d<m\:\:\text{a}\text{n}\text{d}\:d<n$).

From Eq. (1) we can write

3 $$\:Y\:={\Psi\:}\left(\begin{array}{cc}D&\:0\\\:0&\:0\end{array}\right){{\Phi\:}}^{T}$$

For a finite number of initial $\:L$ modes, the truncation criteria are imposed on the singular values as shown below:

4 $$\:\frac{{\sum\:}_{l=L+1}^{r}{\sigma\:}_{l}^{2}}{{\sum\:}_{l=1}^{r}{\sigma\:}_{l}^{2}}\:\le\:\delta\:\:\:$$

where $\:\delta\:$ is a small parameter. So, every mode vector $\:{V}_{j}\:$ may be calculated from the $\:j$th column of $\:\varphi\:$ as

$$\:{V}_{j}=\:\frac{1}{{\sigma\:}_{j}}Y\:{{\Phi\:}}_{j}$$.

Thus, the POD mode matrix can be constructed as below

5 $$\:V=\:\left[{V}_{1}|\:.\:.\:.\:\left|{V}_{j}\right|.\:.\:.\:{|V}_{L}\right]\in\:\:{R}^{\text{m}\times\:\text{L}}$$

Once the POD modes are obtained, they are then used to calculate the projection coefficients ($\:v$) for the snapshot matrix as shown:

$$\:v=\:{V}^{T}Y$$

Similarly, the POD bases and the projection coefficients can be used to find the approximation matrix of Y:

$$\:{Y}^{*}=\:V{V}^{T}Y\:=Vv$$

The quality of the compression/expansion process can be captured by the relative projection error shown in the equation:

6 $$\:{RE}_{POD}=\sum\:_{j=1}^{n}\frac{{\left|\right|{{\left(Y\:\right)}_{j}-\left({Y}^{*}\right)}_{j}\left|\right|}_{2}}{{{\left|\right|\left(Y\:\right)}_{j}\left|\right|}_{2}}$$

where $\:j$ represents the $\:j$^th^ column of the targeted matrix and $\:{\left|\right|.\left|\right|}_{2}$ is the $\:{L}^{2}$- norm.

### Proper orthogonal decomposition and artificial neural network (POD-ANN)

**Fig. 1:**
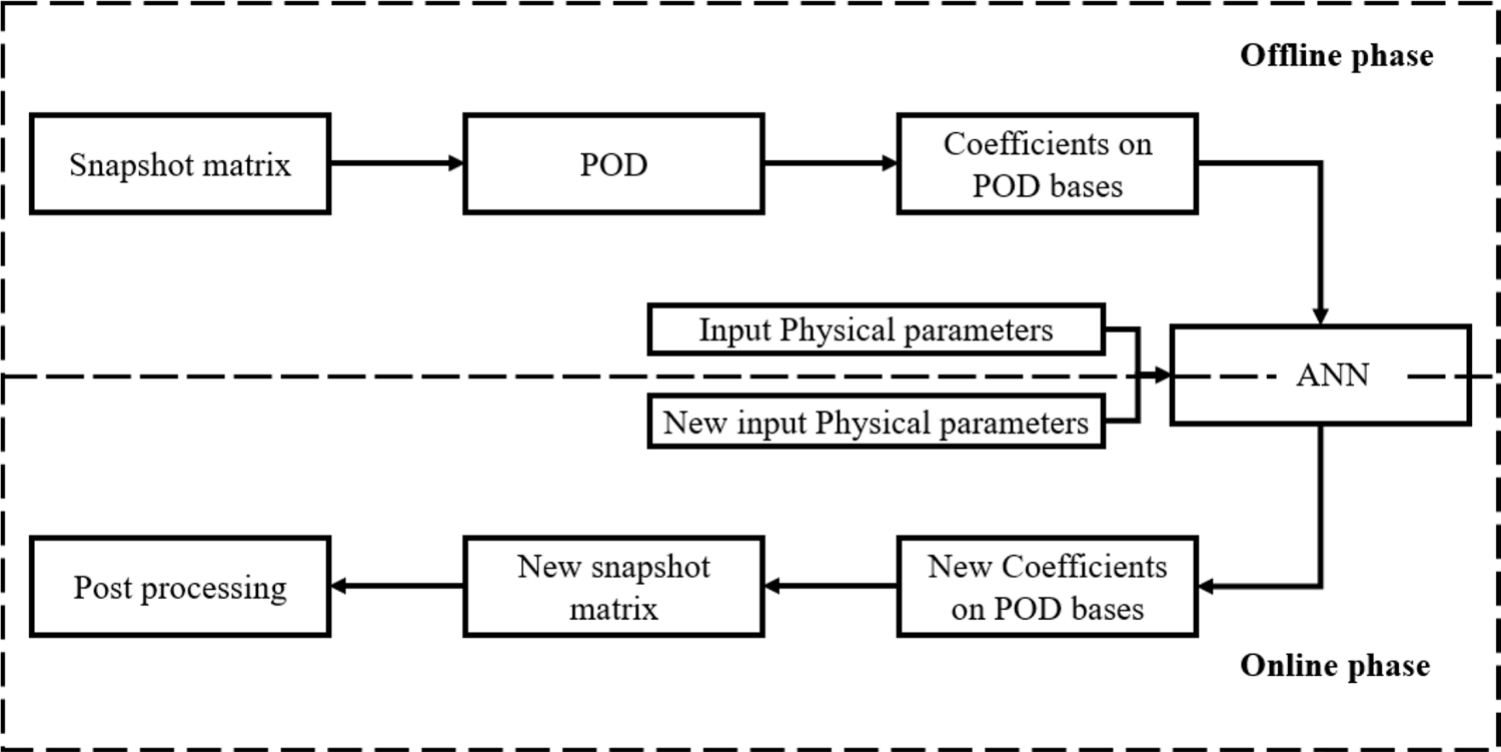
Sketch of the POD-ANN process

A schematic of the POD-ANN method is presented in Fig. 1. In the first step, the POD algorithm extracts the dominant modes from the snapshot matrix. These modes are then used to compute the projection coefficients $\:v$, providing a low-dimensional representation of the original data. Next, an artificial neural network (ANN) is trained to map the input variables to these coefficients $\:v$. The POD-ANN framework consists of two stages: the offline phase, where the ANN is trained using input parameters α and corresponding projection coefficients$\:v$. Here, 80% of the data is allocated for training and 20% for testing.; and the online phase, where the trained ANN predicts new projection coefficients $\:{v}^{\text{*}}$ for a given set of physical parameters ${\rm{ }}{\alpha ^{\rm{*}}}$. These predicted coefficients are then reconstructed into the original high-dimensional space using the process described in Algorithm 1.

**Algorithm 1:**
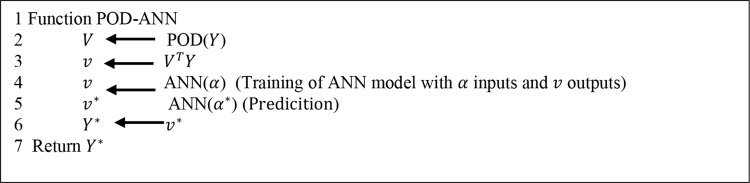
Flowchart of the POD-ANN method

**Fig. 2:**
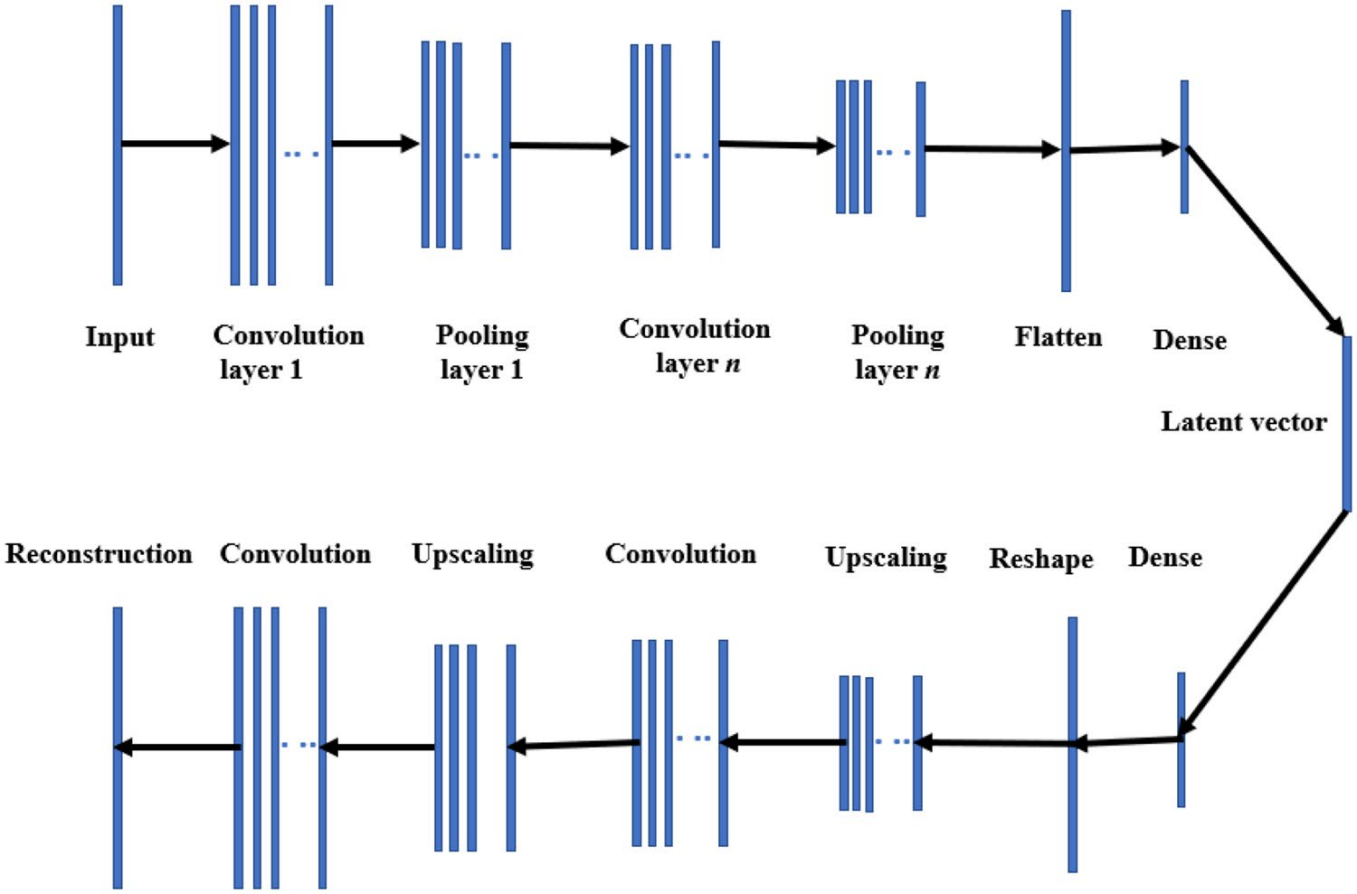
Pictorial representation of a 1D convolution autoencoder architecture

An autoencoder is a type of deep neural network designed for unsupervised feature extraction and dimensionality reduction. Its architecture is well-suited for modal decomposition due to the inclusion of nonlinear activation functions. However, standard autoencoders can struggle to decompose input fields with multiscale coherent features. This limitation can be mitigated by incorporating convolutional layers, which are adept at processing high-dimensional spatial data. Convolutional autoencoders (CAEs) have gained prominence in image recognition and are increasingly applied in nonlinear reduced-order modeling. Unlike classical autoencoders with fully connected layers, CAEs employ convolutional layers to efficiently handle high-dimensional inputs [31]. A CAE consists of two main components: the encoder, which compresses the input matrix into a latent space using convolution, pooling, and dense layers; and the decoder, which reconstructs the original input dimensions from the latent space through upscaling, convolution, and dense layers. The architecture of a CAE is illustrated in Fig. 2. In this study, we adopt a similar CAE structure to encode and decode the spatial dimensions of the input snapshot matrix.

In the convolutional layers, each unit in a feature map is connected to a localized region of the preceding layer through a kernel and activation function. This localized connection allows the network to extract dominant features from the input efficiently. The operation of a 1D convolutional layer can be mathematically expressed as [39]:

7 $$\:{h}_{i}^{l}=\:\sigma\:\left({H}^{l-1}\divideontimes\:{f}_{i}^{l}+{b}_{i}^{l}\right)$$

in which the $\:\divideontimes\:$ denotes the 1D convolution operator, $\:{h}_{i}^{l}\in\:\:{R}^{{D}_{l}\times\:1}$ represents the $\:{i}^{th}$ feature of the $\:{l}^{th}$, $\:\sigma\:$ is the nonlinear activation function, $\:{H}^{l-1}$ = [$\:{h}_{1}^{l-1}$, $\:{h}_{2}^{l-1}$…. $\:{h}_{{{N}_{f}}_{l-1}}^{l-1}$ ] represents the convolution layer $\:l-1$, $\:{b}_{i}^{l}$ gives bias value with $\:i\:\in\:{{N}_{f}}_{l},\:{f}_{i}^{l}$ represents kernel for layer $\:l$ and $\:l\in\:\left(1,n\right).$ The depth of the convolution layer is represented by the total number of layers. After each layer, the pooling layers are inserted to decrease the dimension of the features by an amount that is defined by the kernel size of the pooling layer.

As presented in the previous section, the snapshot matrix consists of a set of $\:n\:$high-fidelity solutions obtained from the numerical simulation$\:\:\left\{y\left({\alpha\:}_{s}\right)\in\:\:{R}^{m}\:,\:\:s=1,\:\dots\:.n\right\}$. In this solver, $\:{\alpha\:}_{s}$ is the $\:{s\:}^{th}$ value of the random variable $\:\alpha\:$ in its data sample with size $\:n$, which follows a probability density function $\:\phi\:\left(\alpha\:\right)$. All these vectors’ solutions are combined and form a global snapshot matrix:

8 $$\text{Y}=\left[{y}_{1}\:..\:.{y}_{s}\dots\:.{y}_{n}\right]\:\in\:\:{R}^{\text{m}\times\:n}\:\:$$

where $\:m$ is the total number of computational nodes in the spatial domain. The global snapshot matrix created above is divided into two sets, one with 80% and the other with 20% of the data for training and testing, respectively.

**Fig. 3:**
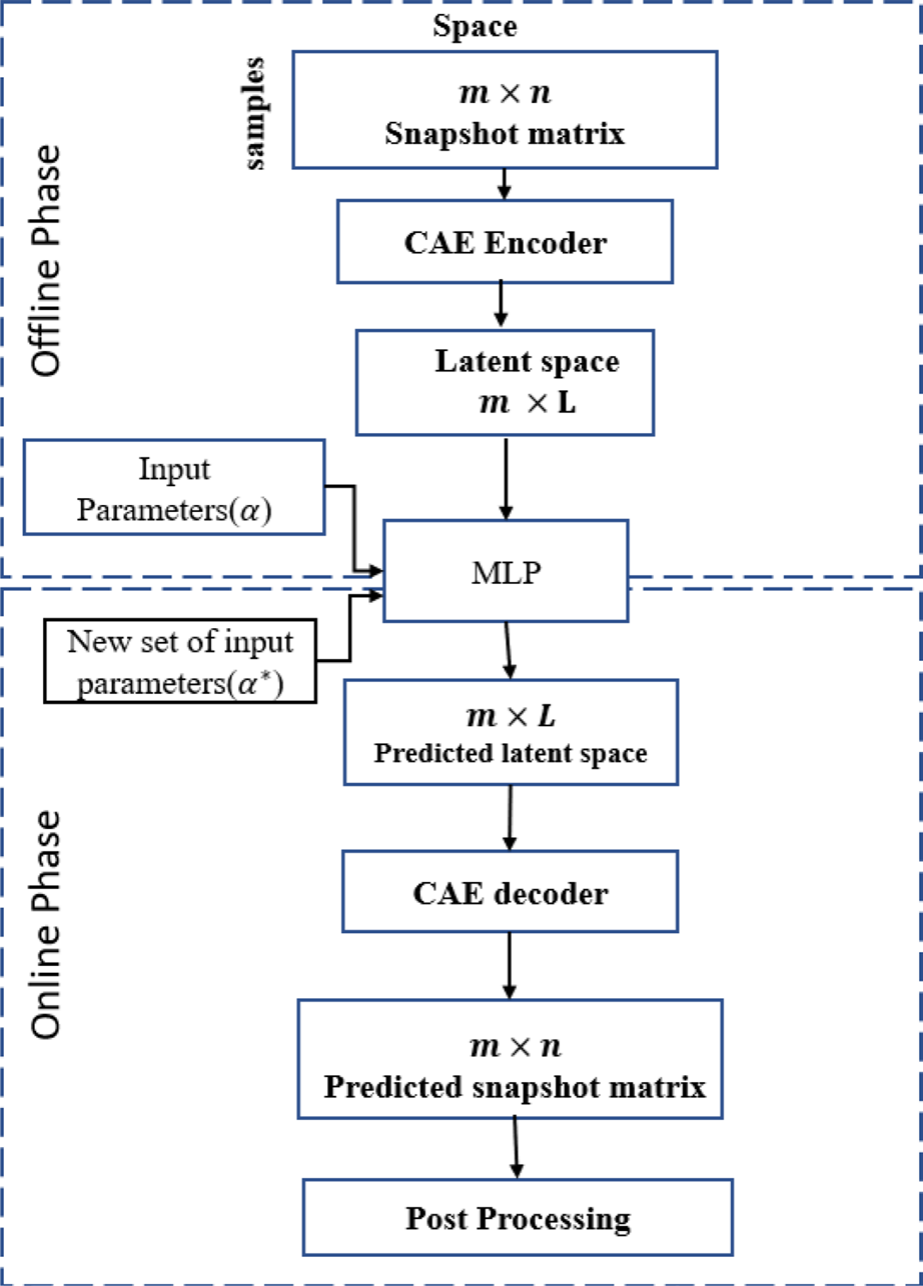
Flow chart of the CAE with its online and offline phases

The CAE framework is divided into three parts: (1) spatial compression, (2) a regression-based multilayer perceptron (MLP), and (3) online surrogate predictions. Spatial compression reduces the dimension of the input data matrix from $\:n$ to $\:L$ along the spatial dimension where $\:L$ represents the latent space dimension. The snapshot matrix $\:\text{Y}$ is reshaped using the space encoder ($\:{{F}_{x}}_{enc}$) part of the CAE along the spatial dimension, as given:

9 $${{V}_{x}}_{L}\:={{F}_{x}}_{enc}\left(\text{Y}\right)\:\:\in\:\:{R}^{\text{m}\times\:\text{L}}$$

where $\:{{V}_{x}}_{L}$ represents the snapshot matrix with reduced dimension. The detailed architectures of each autoencoder used for the test set and the benchmark problem are provided in Appendices Tables A1 and A3. Once the latent space is constructed, the next stage of the CAE framework involves implementing an MLP within this latent space. The MLP model is composed of multiple fully connected layers. Detailed configurations of the MLP applied in each case are given in Appendices Tables A2 and A4. The compression of the spatial dimension using the CAE and the application of the MLP constitute the offline phase of the framework, as illustrated in Fig. 3. It is worth noting that this framework is developed using the open-source package TensorFlow [40] and optimized with the Adam optimizer employing its default parameters. To accelerate optimization and improve convergence during training, the input snapshots are normalized as follows:

10 $$ \widetilde {{u_{si}}}{\rm{ }} = {\rm{ }}{{{Y_{si}} - \min \left( {{Y_s}} \right)} \over {\max \left( {{Y_s}} \right) - \min \left( {{Y_s}} \right)}}{\rm{ s}} = 1 \ldots..{\rm{n}},{\rm{ }}i = 1 \ldots.{\rm{ }}m $$

where $\:{u}_{si}$ is the normalized output for the $\:{\text{s}}^{th}$ input parameter and for the $\:{\text{i}}^{th}$ mesh node.

The final step involves the online surrogate prediction for a new dataset. A new set of input variables is generated using the Latin Hypercube Sampling (LHS) algorithm [41]. For each entry in the new dataset, a spatial latent vector ($\:{{{V}_{x}}_{L}}^{\text{*}}$) is predicted using the trained MLP regression model. This predicted latent space is then decoded back to the original dimensional space using the spatial decoder function ($\:{{F}_{x}}_{dec})$, following the relation:

11 $$\:{Y}^{\text{*}}{={F}_{x}}_{dec}\left({{{V}_{x}}_{L}}^{\text{*}}\right).\:$$

The overall workflow of this framework is illustrated in Fig. 3 and detailed in Algorithm 2.

**Algorithm 2:**
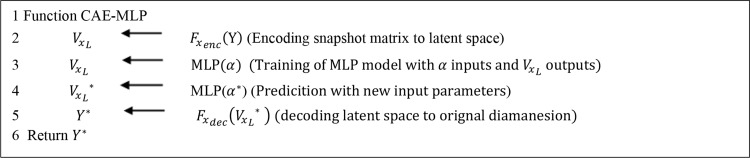
Flowchart of CAE-MLP method

## Results and discussion

This section evaluates the POD-ANN and autoencoder models on two test cases: a 2D heat transfer problem and a 3D additive manufacturing scenario. The results obtained from these reduced-order models are compared with experimental data to demonstrate their efficiency and accuracy.

### 2D heat transfer test case

**Fig. 4:**
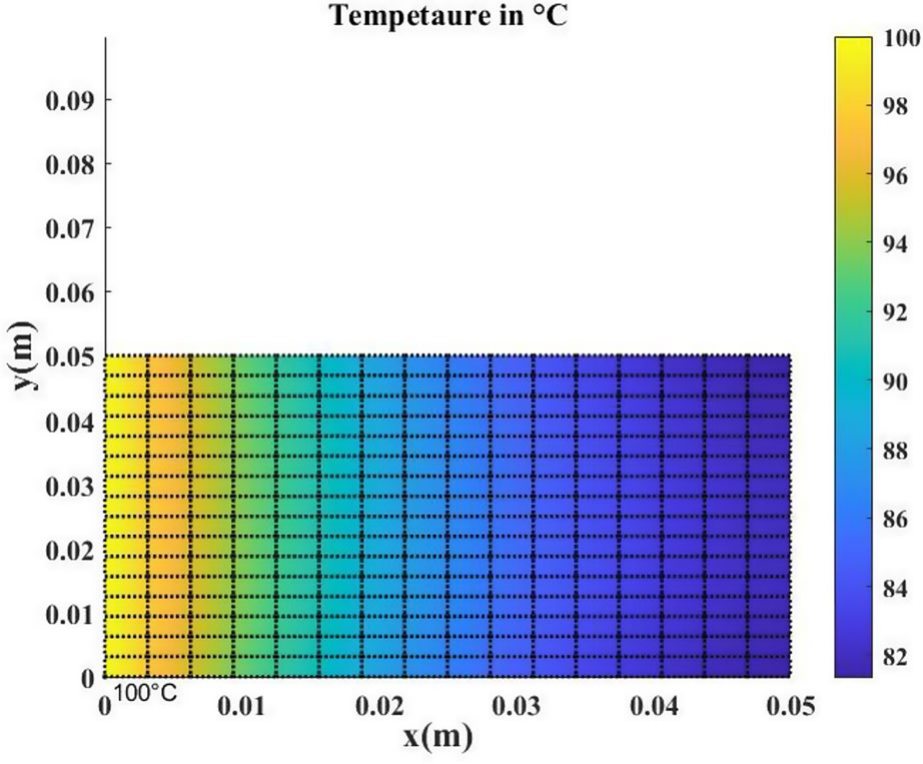
Pictorial representation of the mesh structure and temperature distribution throughout the plate

This preliminary test case investigates steady-state heat transfer through a plate of thickness $\:e$ in a steady state. It serves to validate the implementation of the proposed algorithms. The governing equation and associated boundary conditions are defined by the linear heat equation:

12 $$\:div\:\left(\overrightarrow{q}\right)=\:\frac{2h}{e}\left({T}_{ex}-T\right)$$

where $\:\overrightarrow{q}$ is the conduction flux given by Fourier’s law $\:\overrightarrow{q}=-K\:\nabla\:T$ (K is the conductivity, h is the coefficient of convection and 100°C represents the given temperature on one side. The boundary condition at the edges is $\:\overrightarrow{q}.\overrightarrow{n}=h\left(T-{T}_{ex}\right)$. The temperature is 22 °C at the base (x = 0). In this test case, the coefficients of convection and conductivity are constant and considered input parameters, while the temperature over the entire domain is the output. A total of 300 samples were generated for the input parameters using the Lattice Hypercube Sampling (LHS) algorithm, with samples uniformly collected in the intervals [51, 61] for $\:K$ and [42.6, 57.5] for $\:h$. The heat equation was solved for each input set using an in-house finite element method code. The mesh consists of quadrilateral elements with a total of 289 nodes, as shown in Fig. 4. The output snapshot matrix was used to train the POD-ANN and CAE-MLP models. The dataset of 300 samples was divided into 80% for training and 20% for testing. After training, the predictions from both models with a new set of input parameters were compared with the original snapshot matrix. A set of 5000 samples was generated using the LHS method, and the surrogate models (POD-ANN and CAE) were run to obtain new predictions. A statistical analysis of the outputs was then performed.

**Fig. 5:**
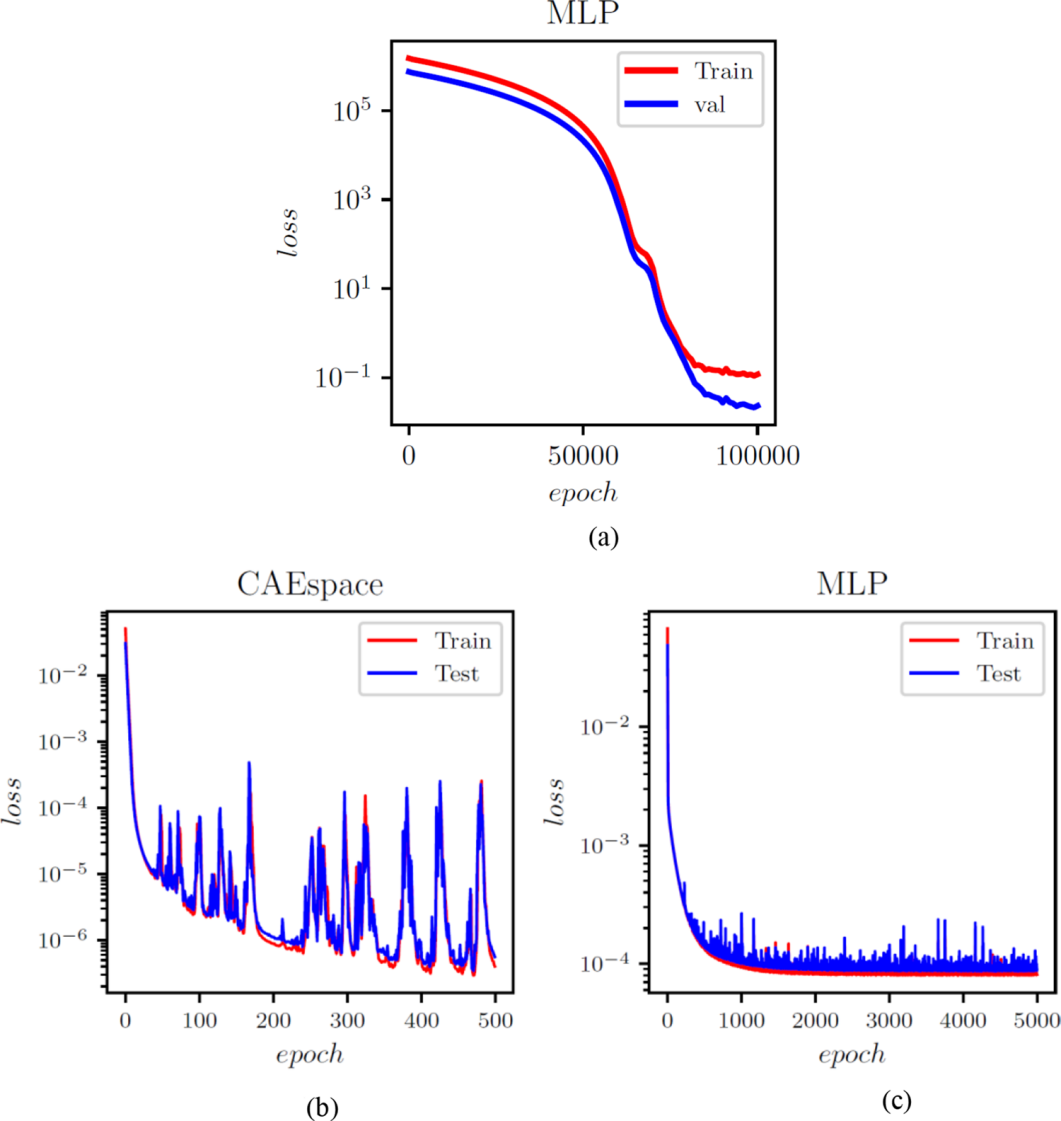
Loss function evolution with epochs for the POD-ANN (a), CAE (b) and the MLP (c) networks

**Fig. 6:**
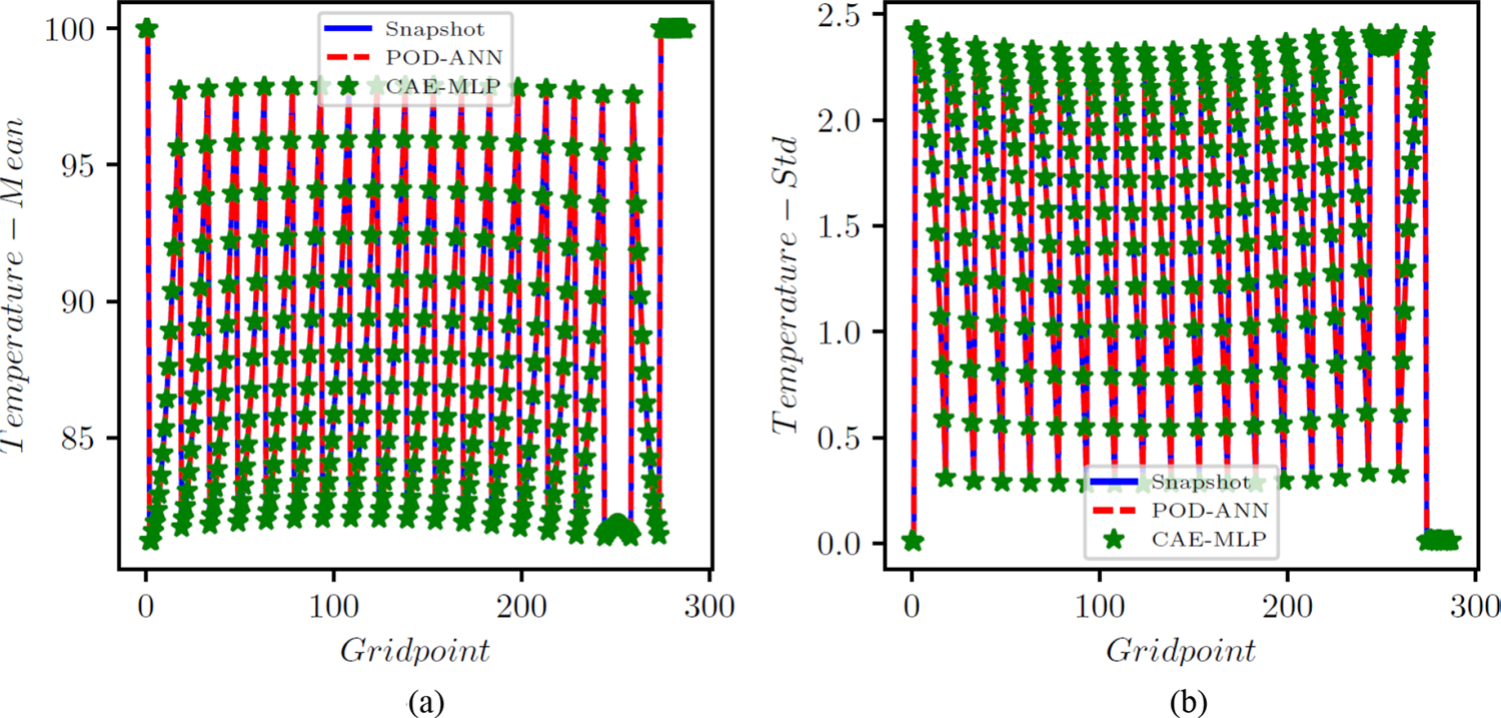
Comparison of the mean (**a**) and std (**b**) of the temperature results between the original snapshot matrix and the temperature predictions of 5000 realizations from POD-ANN and the CAE-MLP networks

The POD-ANN model consists of three hidden layers, each with 50 neurons. The model determined that three POD modes were sufficient to accurately predict the outputs of the heat transfer problem. The CAE-MLP model reduces the spatial dimension from 288 to a latent space dimension of 5. More details on the encoder and decoder structure are provided in Table A1. This process results in a total of 140,587 trainable parameters. The constructed spatial latent space is then mapped to the input variables using a multilayer perceptron (MLP) with 34,053 trainable parameters; the detailed structure is presented in Table A2. The MLP and CAE are trained for 5000 and 500 epochs, respectively, and the loss convergence graphs are shown in Fig. 5. The comparison of the CAE-MLP and POD-ANN models with the snapshot matrix is performed using the standard deviation (std) and the mean of the predicted temperature distribution across the entire domain. The outputs are compared in Fig. 6, which shows a good match between the temperature profiles predicted by the POD-ANN and CAE-MLP models and the original snapshot matrix. The relative L2 error norm between the mean of the predicted temperature profile for the 300-snapshot matrix and each of the 5000 realizations of the POD-ANN and CAE-MLP models are 2.88e-07 and 5.76e-08, respectively. It is important to note that the 5000 realizations are new input parameters, not those used in the training phase. Figure 6(a) shows the mean temperature variation between 100 and 800 °C across the entire domain, while Fig. 6(b) indicates the variation of the temperature profile for each node in the set for a given number of input parameters. Both models required similar times to train and predict the outputs; however, the relative L2 error norm for the CAE-MLP is lower than that of the POD-ANN. This difference indicates that the CAE-MLP predictions are more accurate than those of the POD-ANN. Table 1 provides more details about each model.

**Table 1:** Comparison between the POD-ANN and the CAE-MLP

	POD mode/ Latent space dimension	Training time (sec)	Prediction Time (sec)	Relative L2 error norm (mean)	Relative L2 error norm (std)
POD-ANN	3	422.37	4.15	2.88e -07	9.02e -05
CAE-MLP	5	444.743	5.89	5.76e -08	3.05e -05

### Additive manufacturing benchmark test

**Fig. 7:**
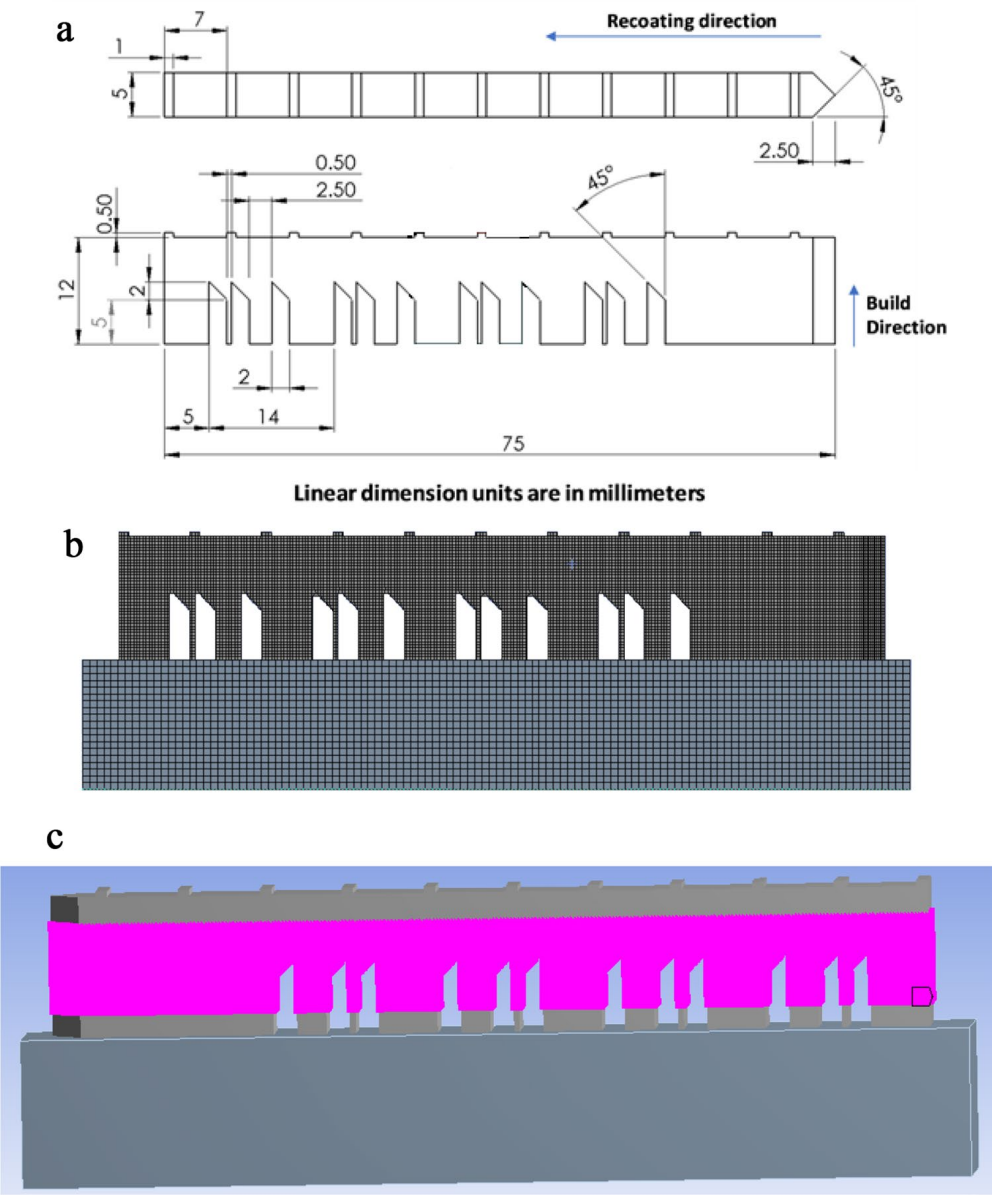
**a** 2D representation of the AMB2018-01 bridge geometry from a plan and an elevation view. **b** Mesh of the bridge and the substrate using workbench additive software. **c** Representation of nodes considered to extract the strain results

In 2018, the National Institute of Standards and Technology (NIST) released several standard benchmark tests for different AM processes to help the community verify their numerical simulations. The AMB2018-01 test is one such case, designed for the selective laser melting process using the material IN625. AMB2018-01 is a bridge structure with dimensions of 75 × 5 × 12.5 mm, built over an 81 × 12.7 × 11 mm substrate, as shown in Fig. 7a and b. The case study aimed to provide reliable data on the residual stresses, strains, and deflections in the built part. The residual stresses and strains were measured using neutron diffraction and x-ray methods, while the deflection was measured after partially cutting the bridge from the base plate. More details on the experimental process and geometry can be found on the NIST website [42]. The additive manufacturing numerical simulation is constructed using the Workbench Additive software. The validation of the workbench model was conducted in a previous publication [4]; for details on the Workbench Additive model, readers are referred to [35]. The simulation took 1 h on 32 CPUs with an Intel E5-2683 v4 processor. The layer thickness, hatch spacing, laser speed, Poisson’s coefficient, and Young’s modulus were considered random input parameters, while the strain across the entire domain was the output variable. The output strains were calculated over 97,360 nodes in the three-dimensional mesh, as shown in Fig. 7c. To eliminate edge or boundary errors, nodes near the boundary were excluded. This simplification reduces the number of nodes, minimizing computation costs.

The five input parameters were selected within the bounds listed in Table 2 to create a dataset of 360 samples randomly chosen using the LHS sampling method. Young’s modulus and Poisson’s ratio were scaled using variables *c*_*1*_​ and *c*_*2*_, respectively, to adjust their values. Normal directional strains over the 97,360 nodes were calculated for each input sample, and the solutions from each sample were combined to construct a high-fidelity snapshot matrix, which was later used to train the POD-ANN and CAE-MLP models.

**Table 2:** Upper and lower bounds for input parameters

Input Parameters	Lower Bound	Upper Bound
laser speed	680 mm/s	920
layer thickness	0.085 mm	0.115
hatch spacing	0.017 mm	0.023
$\:{c}_{1}$Y	0.85	1.15
$\:{c}_{2}$v	0.85	1.15

**Fig. 8:**
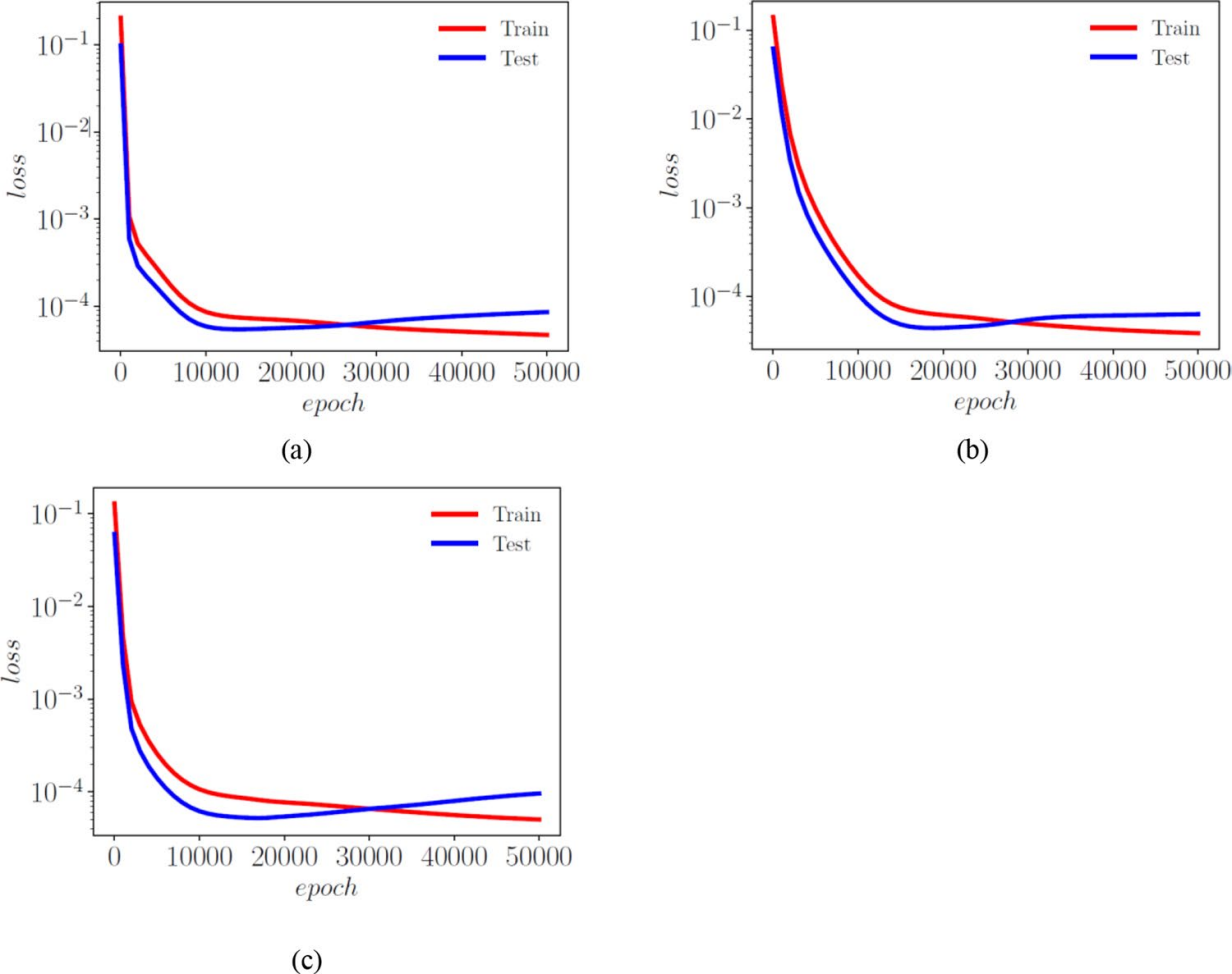
Error graphs for the POD-ANN for the x, y and z strains represented by (**a**), (**b**) and (**c**), respectively

**Fig. 9:**
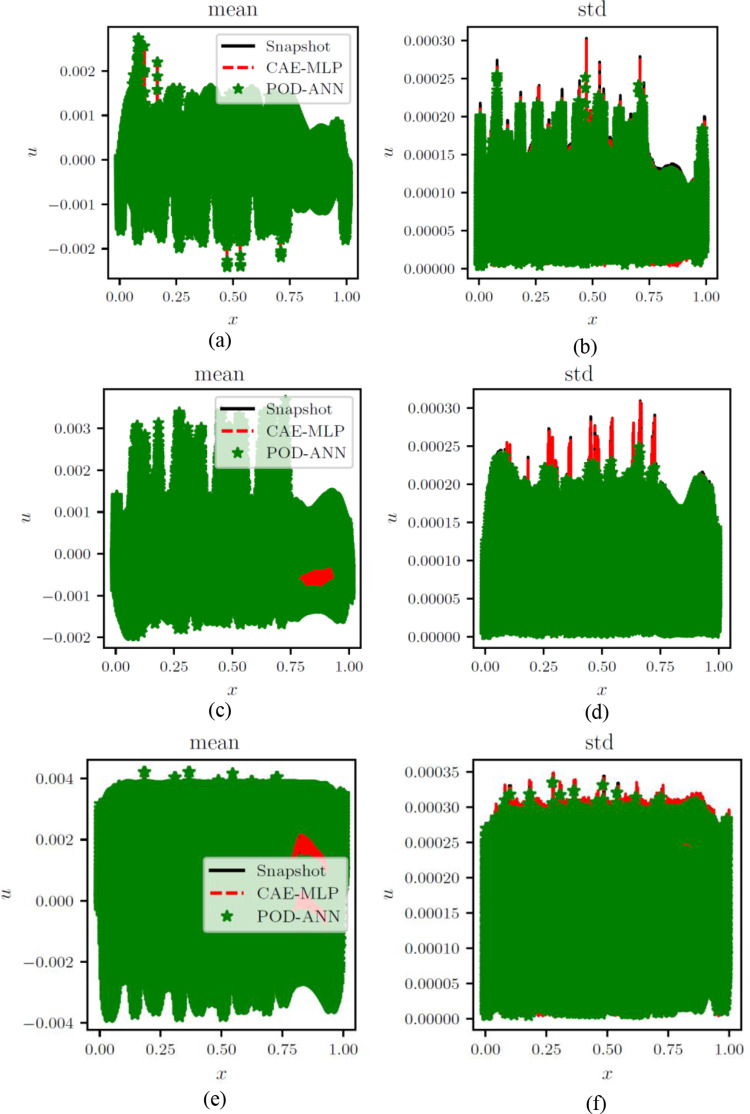
Comparison of POD-ANN and CAE-MLP with the snapshot matrices of the x, y and z strain represented in (**a**-**b**), (**c**-**d**) and (**e**-**f**), respectively, for the whole domain

**Fig. 10:**
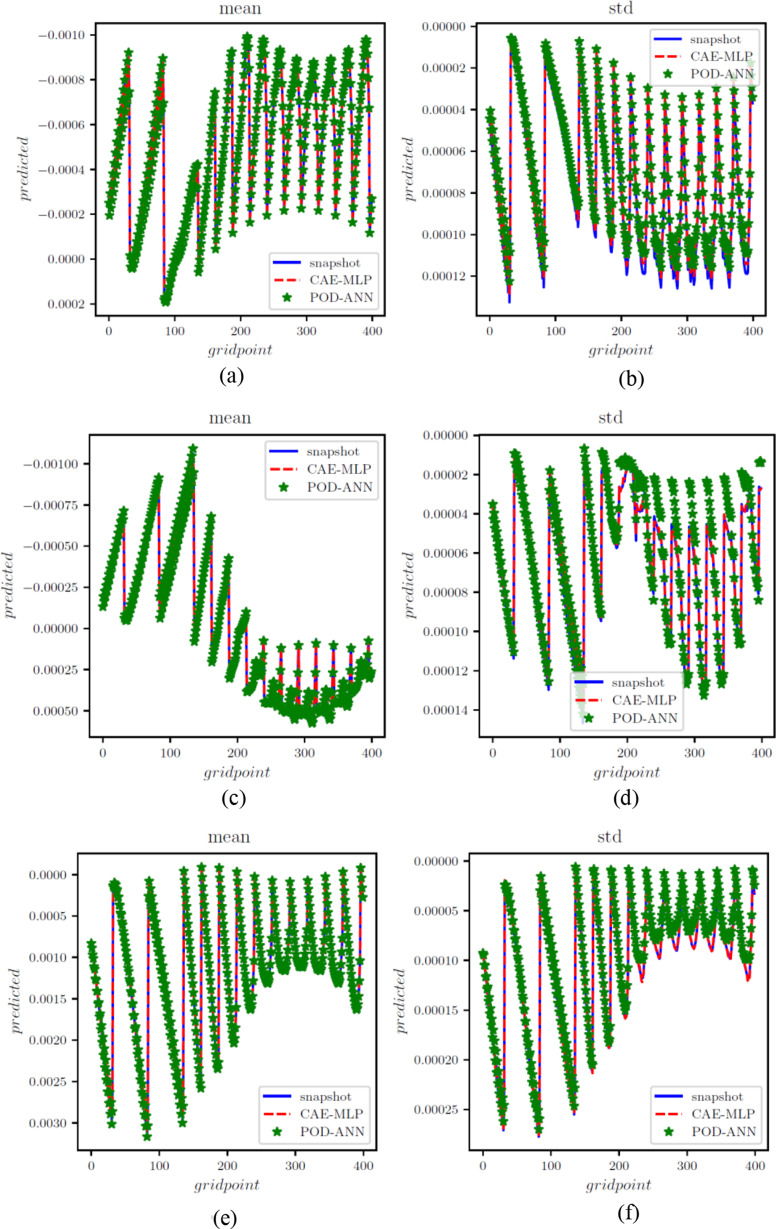
Comparison of POD-ANN and CAE-MLP with the snapshot matrices of the x, y and z strain represented in (**a**-**b**), (**c**-**d**) and (**e**-**f**), respectively, for a cross section

A total of three snapshot matrices, each with dimensions of 360*97,360, were generated for the x, y, and z normal strains. The POD-ANN and CAE-MLP models were trained separately for each matrix. The performance of both models was evaluated by comparing the statistical moments with the original snapshot matrix. Figure 8 shows the training and testing of the POD-ANN models for all three normal strains, while Fig. 9 presents comparisons between the POD-ANN predictions and the snapshot matrix for the entire domain and different directional strains. The POD-ANN uses the Adam optimizer and the ReLU activation function to achieve better results during the learning and prediction phases. The number of POD modes for each normal strain are calculated using the truncation error $\:\delta\:$,which is 10^− 5^, 10^− 6^, and 10^− 7^ for the x, y and z directional strains, respectively. It was determined that 19, 42, and 66 POD modes are sufficient to construct a surrogate model for the given snapshot matrix, as evidenced by the error graph in Fig. 8. Figure 9 demonstrates that the mean and standard deviation results correlate well with the original snapshot matrix. The strain graphs indicate that the body has undergone compression in certain areas and expansion in others. The comparison between the mean and standard deviation results suggests that the predictions are accurate. This model can be utilized to calculate the strain at any given point in the geometry and for unseen input parameters within the training intervals. The results along a random cross-section of the bridge are provided in Fig. 10. Additional details related to the POD-ANN structure and effects are presented in Table 3.

**Table 3:** POD_ANN structure

	POD mode	Hidden layers of ANN	Number of neurons in each layer	Training time (Sec)	Prediction time (Sec)	Relative L2 error mean	Relative L2 error std
X_ strain	19	4	100	413.024	40.96	0.0003725	0.0148
Y_ strain	42	5	30	226.7586	39.6269	0.0000698	0.00871
Z_ strain	66	5	50	290.95	37.41	1.898 e-05	0.0040

**Fig. B1:**
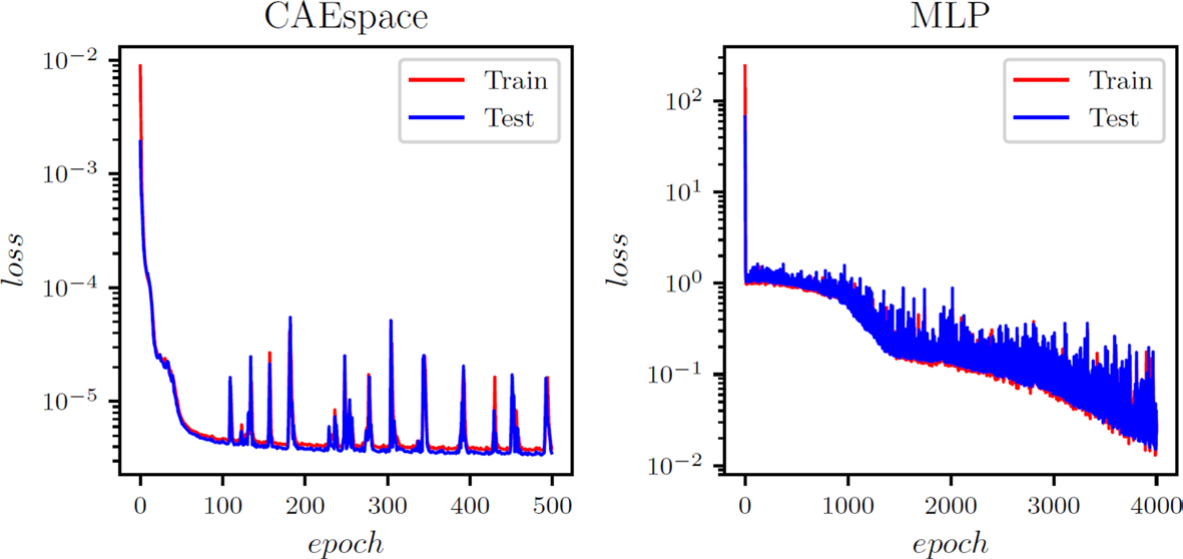
Evolution of the training and validation error for the x-strain in the AMB2018-01 benchmark case

**Fig. B2:**
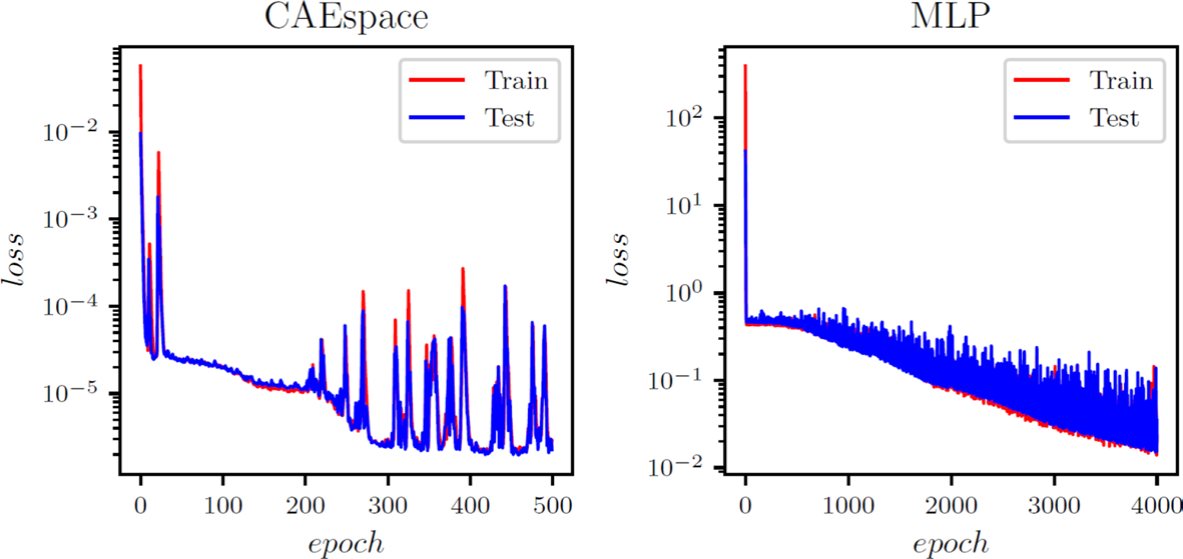
Evolution of the training and validation error for the y -strain in the AMB2018-01 benchmark case

**Fig. B3:**
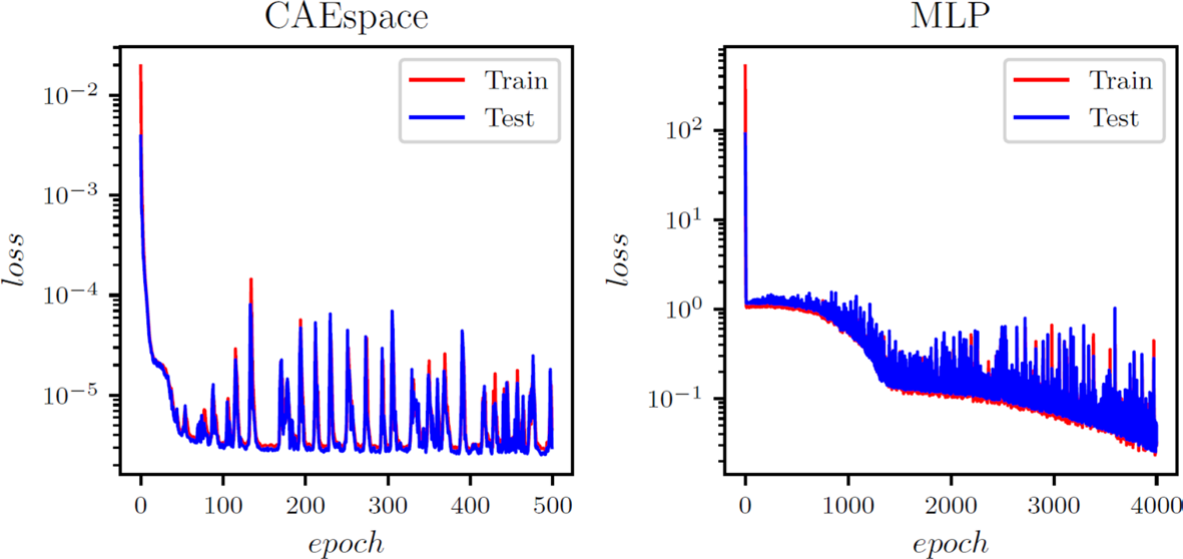
Evolution of the training and validation error for the z-strain in the AMB2018-01 benchmark case

Similar to the heat transfer test case, the spatial autoencoder structure consists of 1D convolution layers with 10 and 15 channels, along with max pooling layers and a non-linear activation function (ReLU). These components help reduce the spatial dimension from 97,360 to 19, 40, and 60 for the x, y, and z directional strains, respectively. The reduced dimensions represent the spatial latent space and are directly connected to the input parameters via an MLP model. Figure 3 illustrates the working flowchart of the CAE-MLP model, while the detailed architecture of the CAE-spatial and MLP is presented in Appendix Table A3 A and A4 A. During the training process, the CAE is trained for 500 epochs, followed by the MLP for 1,000 epochs. The convergence history of the CAE and MLP for the x, y, and z strains is shown in Figures B1-B3 in the appendix. he CAE-MLP model is trained on the original snapshot matrices for x, y, and z separately, with the calculated outputs presented in terms of standard deviation and mean. Similar to the POD-ANN process, the trained CAE-MLP model is used to predict outputs for a new dataset of 5,000 samples, and the variations in the statistical moments are compared with the original snapshot matrices for each normal strain. Figures 9 and 10 illustrate the variations in the CAE-MLP predictions for the entire domain and a random cross-section of AMB2018-01, respectively, to better visualize the results. The results from the CAE-MLP are also compared with those of the POD-ANN in Figs. 9 and 10 and listed in Table 4. The complete architecture of the CAE-MLP model for different strains is shown in Appendix Table 3 A and 4 A. Both models are trained with 80% of the initial snapshot matrix, while the remaining 20% is used to test the models. Compared to the POD-ANN, the CAE-MLP model takes more time to train and predict, but the CAE-MLP predictions are more accurate. The L2 norm between the statistical moments of CAE-MLP predictions and the snapshot matrix is lower than that of the POD-ANN predictions for all strains. Table 4 lists the results of both models for easy comparison.

**Table 4:** Comparison of POD-ANN and CAE-MLP results

	Modes	Relative L2 error for mean	Relative L2 error for std
POD-ANN x strain	19	0.00037	0.0149
CAE-MLP x strain	19	0.0001	0.0038
POD-ANN y strain	44	0.0006	0.0088
CAE-MLP y strain	40	0.0001	0.0014
POD-ANN z strain	66	0.000017	0.0039
CAE-MLP z strain	60	0.0002	0.00049

### Comparison with experimental results

**Fig. 11:**
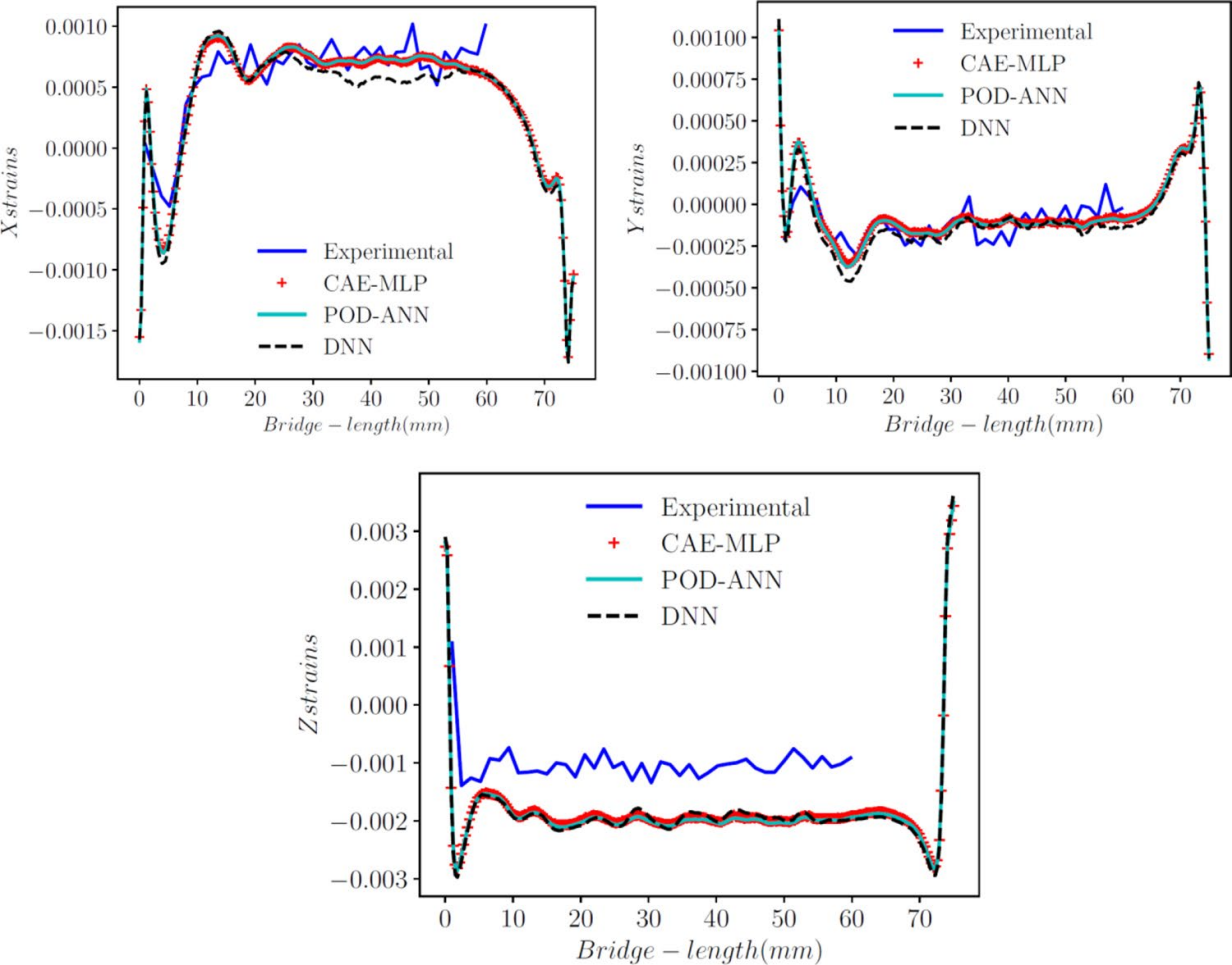
Comparison of the POD-ANN and CAE-MLP models with the experimental results for normal strains in the x, y, and z directions

This section compares both models and the experimental outputs. The means of the 5,000 sample outputs from both models are compared with the experimental data at location z = 9.536, as shown in Fig. 11. These outcomes are also compared with the outputs generated from the DNN model in [4] over the same cross-section. Chaudhry et al. [4] constructed a surrogate model using the DNN model for the normal strains at the experimental location points.

z = 9.536 with only 251 nodes. Comparing the three models indicates that the reduced-order models constructed over the whole geometry provide better results than the DNN model built with just a cross-section. The new approaches improve the accuracy of the results by 60%-80%. The CAE-MLP offered significantly better results than the DNN and even improved on the POD-ANN, providing better results for all three normal strains. The relative L2​ norm values for the models and the experimental data are presented in Table 5 for each directional strain.

**Fig. 12:**
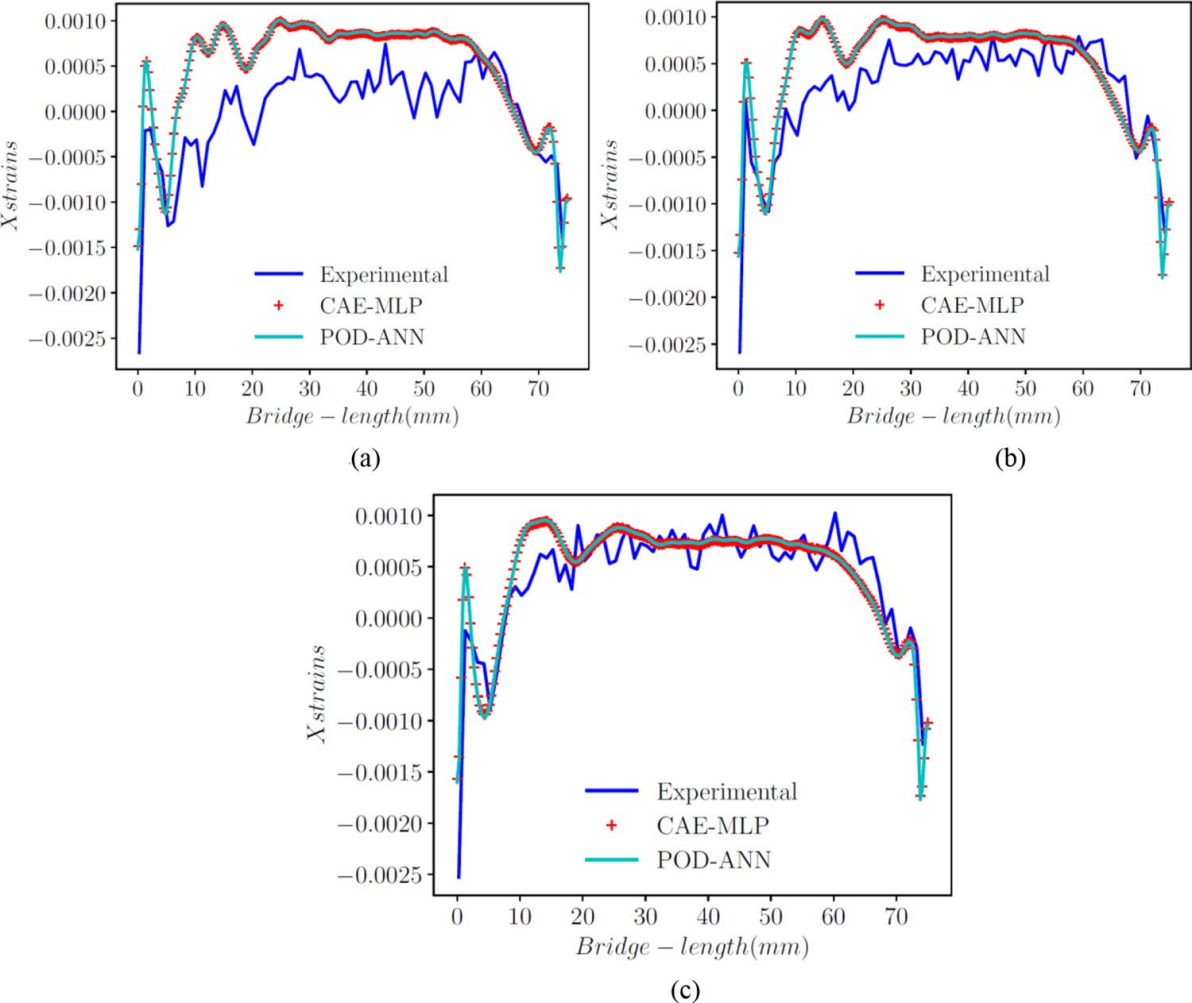
Comparison of the x strains found with the POD-ANN and CAE-MLP models with the experimental results at z = 8.25, 8.75 and 9.25, in (**a**), (**b**) and (**c**), respectively

Additionally, the prediction results for normal directions with the POD-ANN and CAE-MLP are compared with the experimental results at three different locations. Figure 12 shows the good correlation between the experimental outputs and the predictions at z = 8.25 mm, 8.75 mm, and 9.25 mm. A total of 40 experimental data points for each location were considered and compared up to a length of 60 mm of the bridge. These results demonstrate the ability of the proposed technique to accurately predict strain values across the entire domain.

**Table 5:** Comparison of relative L2 error norm values of 3 ML approaches with the experimental results

	POD-ANN	CAE-MLP	DNN
X strain	0.040	0.039	0.271
Y strain	0.234	0.212	0.658
Z strain	0.789	0.74	0.873

## Conclusion

This study introduced non-intrusive reduced-order models—POD-ANN and CAE-MLP—to analyze the selective laser melting (SLM) process. By leveraging proper orthogonal decomposition and deep convolutional autoencoders, the proposed frameworks demonstrated both efficiency and effectiveness in constructing data-driven surrogate models that combine techniques from reduced-order modeling and machine learning.

Both approaches operate in two stages: an offline phase and an online phase. During the offline phase, a high-fidelity snapshot matrix (97,360 × 360), representing normal strains computed using Workbench Additive software, is used. In this stage, the POD-ANN employs proper orthogonal decomposition to reduce the dimensionality of the snapshot matrix, while the CAE-MLP utilizes an autoencoder to encode the spatial dimension into a latent space. The decoder subsequently reconstructs the original dimension from the latent representation. The reduced latent space is mapped to the input variables using a multilayer perceptron (MLP). In the online phase, new input datasets are provided, and the trained models predict the corresponding outputs. For CAE-MLP, the predicted latent vectors are decoded back into the original spatial dimension, enabling reconstruction of the strain fields.

The efficiency and accuracy of these approaches were validated on two test cases: a steady-state heat transfer problem and the AMB2018-01 SLM benchmark from NIST. Both models achieved excellent agreement with high-fidelity simulations within ± 15% variation of input parameters. In the benchmark SLM case, CAE-MLP provided a superior approximation of statistical moments relative to the original snapshot matrix and outperformed POD-ANN in modeling nonlinear complexities. The CAE-MLP exhibited lower relative error norms and demonstrated enhanced capability for representing intricate physical phenomena.

Moreover, predictions from both models were compared to experimental data and results from a deep neural network (DNN) model reported in [40]. Notably, both POD-ANN and CAE-MLP showed improved correlation with experimental observations, with CAE-MLP achieving prediction accuracy gains of 60–80% over the prior DNN approach. These findings underscore the potential of the proposed methods for generating high-fidelity surrogates capable of capturing the essential dynamics of additive manufacturing processes.

While these models exhibit promising performance, their reliance on training data limits generalization beyond the sampled parameter space. Future work will focus on incorporating uncertainty quantification techniques—such as ensemble methods or Bayesian neural networks [43–45], to provide confidence intervals, enhance robustness, and ensure reliability in extrapolative scenarios. Such developments will make these reduced-order models even more applicable to real-world industrial applications where variability and unknowns are inevitable. Furthermore, recent advances in Physics-Informed Neural Networks (PINNs) for additive manufacturing—including thermomechanically augmented PINNs for stress prediction [19] and transfer learning-enhanced PINNs for melt pool morphology estimation [25] suggest promising avenues for hybrid approaches. Combining physics-based constraints with data-driven ROMs could enhance generalization, interpretability, and reliability, paving the way for their deployment in real-world SLM workflows and optimization pipelines.

Thus, the proposed POD-ANN and CAE-MLP models present powerful tools for studying highly nonlinear, complex physical systems by reducing computational costs without sacrificing accuracy. They offer a compelling foundation for advancing surrogate modeling in additive manufacturing and beyond.

## Appendix A

This section provides the convergence history and the architecture for the CAE-MLP and POD-ANN models.

Table A.1: CAE space architecture for the heat transfer test case:

**Table A.1a:** Encoder-Space

	Filters	Activation function	Kernel shape
Input	-	-	-
Conv-pooling	32	Prelu	3 × 2
Conv-pooling	68	Prelu	3 × 2
Conv-pooling	128	Prelu	3 × 2
Flatten	-	-	-
Dense	-	Prelu	-
Dense (output -$\:{L}_{x}$)	-	Prelu	-

**Table A.1b:** Decoder- space

	Filters	Activation function	Kernel shape
Input ($\:{L}_{x}$)	-	-	-
Dense	-	Prelu	-
Reshape	-	Prelu	-
Conv-upsamp	128	Prelu	3 × 2
Conv-upsamp	68	Prelu	3 × 2
Conv-upsamp	32	Prelu	3 × 2
Output	1	Prelu	3

**Table A.2:** MLP architecture for heat transfer case

Layer type	Output size	Activation function
Input layer	2	Prelu
Dense layer	128	Prelu
Dense layer	128	Prelu
Dense layer	128	Prelu
Output layer	$\:{L}_{x}=5$	Linear

Table A3: CAE space architecture for the AMB2018-01 benchmark case:

**Table A 3a:** Encoder-Space

	Filter size	Kernel shape	Activation function
Input	-	-	-
Con-pooling	10	3 × 2	Prelu
Con-pooling	15	3 × 2	Prelu
Flatten	-	-	-
Dense	-	-	Prelu
Dense (output -$\:{L}_{x}$)	-	-	Prelu

**Table A.3b:** Decoder- Space

	Filter size	Kernel shape	Activation function
Input ($\:{L}_{x}$)	-	-	-
Dense	-	-	Prelu
Reshape	-	-	Prelu
Con-upsam	15	3 × 2	Prelu
Con-upsam	10	3 × 2	Prelu
Output layer	1	3	Prelu

**Table A.4:** MLP architecture for the AMB2018-01 cases

Layer type	Output size	Activation function
Input layer	5	Prelu
Dense layer	128	Prelu
Dense layer	128	Prelu
Dense layer	128	Prelu
Output layer	$\:{L}_{x}=\text{19,40,60}$	Linear

## Data Availability

Data cannot be shared openly but are available on request from authors.
